# Supplementary material for: ganon2: up-to-date and scalable metagenomics analysis
Source: NAR Genom Bioinform. 2025 Jul 17;7(3):lqaf094. doi: 10.1093/nargab/lqaf094 (PMC12267982; doi:10.1093/nargab/lqaf094)
Supplement: lqaf094_Supplemental_Files [file lqaf094_supplemental_files.zip › ganon2_benchmark.html]

MetaBench
