# Supplementary material for: ganon2: up-to-date and scalable metagenomics analysis
Source: NAR Genom Bioinform. 2025 Jul 17;7(3):lqaf094. doi: 10.1093/nargab/lqaf094 (PMC12267982; doi:10.1093/nargab/lqaf094)
Supplement: lqaf094_Supplemental_Files [file lqaf094_supplemental_files.zip › SupplementaryFigures.pdf]

ganon2: up-to-date and scalable metagenomics analysis
Supplementary Figures

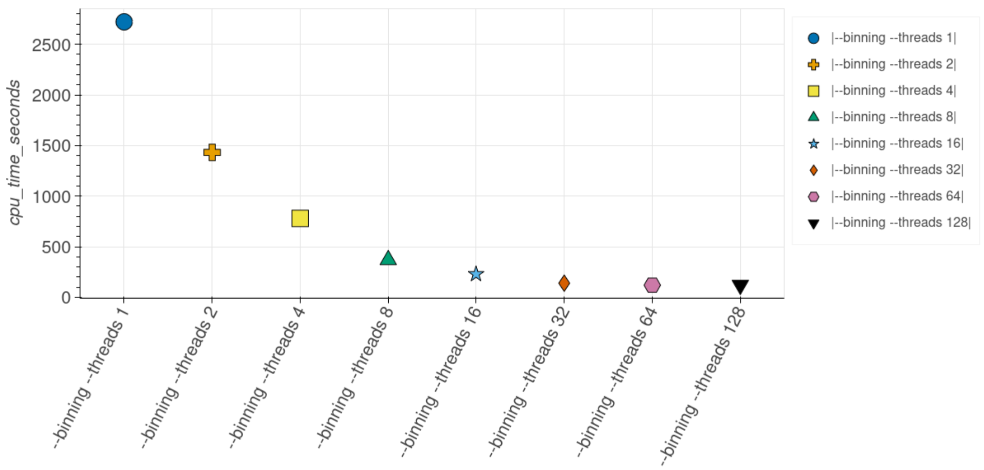

Supplementary Figure 1 Time (seconds) to profile one CAMI2 Challenge Marine sample against the RefSeq CG+RG with different number of threads. Each parametrization was executed 3 consecutive times and only the fastest was considered.
